# Clinical prediction model for red cell blood transfusion in elective primary posterior lumbar spine fusion

**DOI:** 10.1038/s41598-024-65174-2

**Published:** 2024-06-21

**Authors:** Chatchawan Pirot, Chakkraphan Tantrakansakun, Todsaporn Sirithiantong

**Affiliations:** Department of Orthopaedics, Hatyai Hospital, Songkhla, 90110 Thailand

**Keywords:** Health care, Medical research, Risk factors

## Abstract

Overestimated the cross-match of preoperative PRC preparation for elective primary lumbar spinal fusion needs revision for cost-effectiveness. We aimed to develop a novel preoperative predictive model for appropriate PRC preparation. This clinical prediction model in a retrospective cohort was studied between January 2015 and September 2022. Multivariate logistic regression models were used to assess predictive variables. The logistic coefficient of each predictor generated scores to establish a predictive model. The area under the receiver operating characteristic curve (AuROC) was used to evaluate the model. The predictive performance was validated using bootstrapping techniques and externally validated in 102 independent cases. Among 416 patients, 178 (43%) required transfusion. Four final predictors: preoperative hematocrit level, laminectomy level, transforaminal lumbar interbody fusion level, and sacral fusion. When categorized into two risk groups, the positive predictive values for the low-risk score (≤ 4) were 18.4 (95% Cl 13.9, 23.6) and 83.9 (95% CI 77.1, 89.3) for the high-risk score (> 4). AuROC was 0.90. Internal validation (bootstrap shrinkage = 0.993) and external validation (AuROC: 0.91). A new model demonstrated exemplary performance and discrimination in predicting the appropriate preparation for PRC. This study should be corroborated by rigorous external validation in other hospitals and by prospective assessments.

## Introduction

Elective primary lumbar spine fusion is a major surgery with a high risk of perioperative blood loss associated with increased blood component transfusion requirements. Significant blood loss^1^ requires a packed red cell (PRC) transfusion of approximately 50–81%^2^. A systematic review^2^ revealed significant postoperative cardiac and noncardiac complications, such as surgical site infection, deep vein thrombosis, pulmonary embolism, myocardial infarction, transient ischemic attack, stroke, respiratory tract infection, and sepsis, in allogeneic transfusion. A prospective randomized controlled trial revealed that preoperative autologous blood donation reduces the risk of allogeneic blood transfusion in patients who undergo elective lumbar spine surgery^3^. The preoperative cross-matched transfusion ratio (C:T ratio) was overestimated. The high C:T ratio results in the loss of global costs in the management chain of blood processes, such as blood bank resources, time, finances, and human resources^4–6^. As recommended, cross-match PRC by the maximum surgical blood-order schedule (MSBOS) was indicated for general preparation of PRC in lumbar spine surgery^7^.

Previous potential predictors associated with the risk of PRC transfusion may guide the general adjustment for the cross-match order, such as female sex^8–10^, older age^8,9^, high body mass index (BMI)^1^, pulmonary disease or dyspnea^8,9,11^, bleeding disorders^8^, anticoagulant/antiplatelet therapy^8^, high American Society of Anesthesiologist (ASA) classification^1,9,12^, low preoperative hemoglobin (Hb) levels^11^, hematocrit (Hct)^8,9^, multilevel surgery (laminectomy and fusion)^8,9,11–13^, long surgical time^8,9,11–13^, transforaminal lumbar interbody fusion (TLIF)^1,12^, and sacrum fusion^12^. Recent limited studies^14^ revealed that a nanogram for PRC transfusion was not simplified for application, reported only preoperative predictors^15^, and did not define the type of fusion^3^. Intraoperative procedures were strong predictors that affected the accuracy of the prediction model^1,3,8,9,11–14,16^, but they were inappropriate in the preoperative prediction model. Lumbar spine magnetic resonance imaging stimulated preoperative procedure planning in a previous cohort^17^, similar to actual surgery. This study used preoperative procedural planning in this model.

They overestimated the cross-match PRC, which resulted in a blood reservation shortage, especially during the coronavirus disease 2019 pandemic^18^. The MSBOS recommends a general cross-match PRC of two units for lumbar spine surgery^19^. PRC transfusions in this spine referral center demonstrated a 43% prevalence. To date, limited data is available regarding the influencing factors in determining an appropriate PRC transfusion for elective primary lumbar spine fusion in developing countries, where healthcare resources are relatively limited. Additionally, the parameters for predicting the probability of PRC transfusion have no practical use in surgical planning. Geographic variations in healthcare resources, socioeconomic status, and ethnicity may affect predictive PRC preparation. This study aimed to develop a preoperative predictive model for appropriate PRC transfusion in elective primary lumbar spine fusion.

## Materials and methods

### Study design and population

A retrospective observational cohort design and prognostic prediction model were developed using data from a spine referral center hospital. The Institutional Ethics Committee approved the study protocol, which was conducted in accordance with the Declaration of Helsinki.

### Selection of participants

This study included patients aged ≥ 50 years who underwent elective primary posterior lumbar spine fusion. The inclusion criteria were: (1) Lumbar disc herniation, (2) Lumbar spinal stenosis, (3) Lumbar spondylolisthesis, and (4) Lumbar disc herniation with spinal stenosis at a tertiary spine referral center. Exclusion criteria were (1) trauma and emergencies, (2) minimally invasive or endoscopic techniques, (3) tumors, (4) infection, (5) revision spine surgery, and (6) thoracic and cervical levels. The electronic medical records between January 2015 and September 2022 were retrospectively analyzed.

### Data collection

Potential clinical predictors include baseline characteristics, such as female sex^8–10^, age^8,9^, and BMI^1^; comorbidities, such as type II diabetes mellitus^8,11^, hypertension^11^, pulmonary disease^3,8,9^, anticoagulant or antiplatelet^8^, ASA classification^1,9,12^; preoperative laboratory parameters, such as preoperative Hct^8,9^ and platelets^14^; operative data, such as operative time^8,9,11–13^, decompression level and fusion method^1,8,9,11–13^, sacral fusion^12^, number of pedicular screw fixations^3^, use of tranexamic acid^20^, and estimated blood loss (EBL)^14^.

This study categorized the intraoperative transfusion of PRC into the transfusion and nontransfusion groups.

### Sample size calculation

No standard recommended approach has been used for sample size calculations in the development of clinical prediction models. A database was used for score derivation to maximize statistical power and generalizability. The minimum sample size required to develop a multivariable prediction based on the rule of thumb to estimate the sample size used for a prediction model in the 1990s included ≥ 10 events per predictor^21^.

### Ethical approval

The study protocol was approved by the Institutional Ethics Committee of Hatyai Hospital (Protocol no. HYH EC 085-65-01) and was conducted in accordance with the Declaration of Helsinki.

### Informed consent

A comprehensive agreement for academic use of information acquired during their treatments was obtained from the patients by the hospital at the time of their hospitalization, and no identifiable information of the participants is included in this manuscript.

## Statistical methods

### Statistical analysis

Continuous data are presented as the mean and standard deviation (SD), and categorical data are presented as frequencies and percentages. Comparisons of categorical data were performed using the chi-square test or Fisher’s exact probability test, and unpaired t-tests were used for continuous data. Variables significant in the univariate logistic regression were subsequently included in the multivariable logistic regression analyses using STATA version 15.1 (Stata et al. Station, TX, USA). Statistical significance was set at *P* < 0.05.

### Model development

Eliminating each of the 19 candidate predictors depends on the magnitude of association (odds ratio), statistical significance (*P*-value), AuROC, or significant clinical-related predictors. Logistic regression analysis was used to identify predictors of PRC transfusion. First, univariate analysis was used to analyze the baseline characteristics, comorbidities, preoperative laboratory findings, and operative data. This model avoided bias; significance predictors from univariate analysis were only determined once they were considered in the multivariable model^22^. Significant variables (*P* < 0.05) were then included in a multiple logistic regression model with backward selection. The reduced multivariable model retained its predictive performance in terms of discrimination and calibration, and clinical AuROC was used to evaluate the discriminative ability of the derived score. Calibration using the calibration curve and Hosmer–Lemeshow goodness-of-fit test, where a nonsignificant χ^2^ value indicates a good fit model. The decision curve analysis determined the potential clinical use, which calculates the net benefit of using the model in practice to classify patients across a range of clinically relevant threshold probabilities compared with transfusion and non transfusion of PRC in patients with elective primary posterior lumbar spine fusion. Each model’s performance included sensitivity, specificity, positive predictive value (PPV), and negative predictive value (NPV).

The final predictors were assigned the logistic regression coefficients. After model reduction, the regression coefficients, in log-odds form, of the remaining predictors were determined and used to generate a weighted score. The model’s lowest coefficient was categorized by dividing each predictor’s logistic coefficient and then rounded to the nearest nondecimal integer for applicability. Classification of the sum score indicated a lower or higher risk. The calculated PPV was assigned to each score group to indicate the average patient predictor. Measures of calibration and discrimination were also performed using regression with the PRC transfusion on the model. A calibration plot comparing the model-predicted risk with the observed risk indicated predictive performance. Internally validated by nonparametric receiver operating characteristic (ROC) regression with 1,000 bootstrapped replicates and externally validated in 102 independent cases. Statistical significance was set at p < 0.05.

Scores were classified into two risk groups for clinical utility: low and high-risk. In the low-risk group, lower cut-off points minimized the magnitude of the PPV, while higher cut-off points maximized the magnitude of the PPV in the high-risk group. The model’s discriminative ability used 95% CIs to avoid overlapping with the specific PPV. The potential clinical use of the model was identified by decision curve analysis, which calculates the net benefit of applying the model to classify patients across a range of clinically relevant threshold probabilities compared to the two groups of outcomes (transfusion or non transfusion of PRC) in patients with elective primary posterior lumbar spine fusion.

## Results

**Figure 1:**
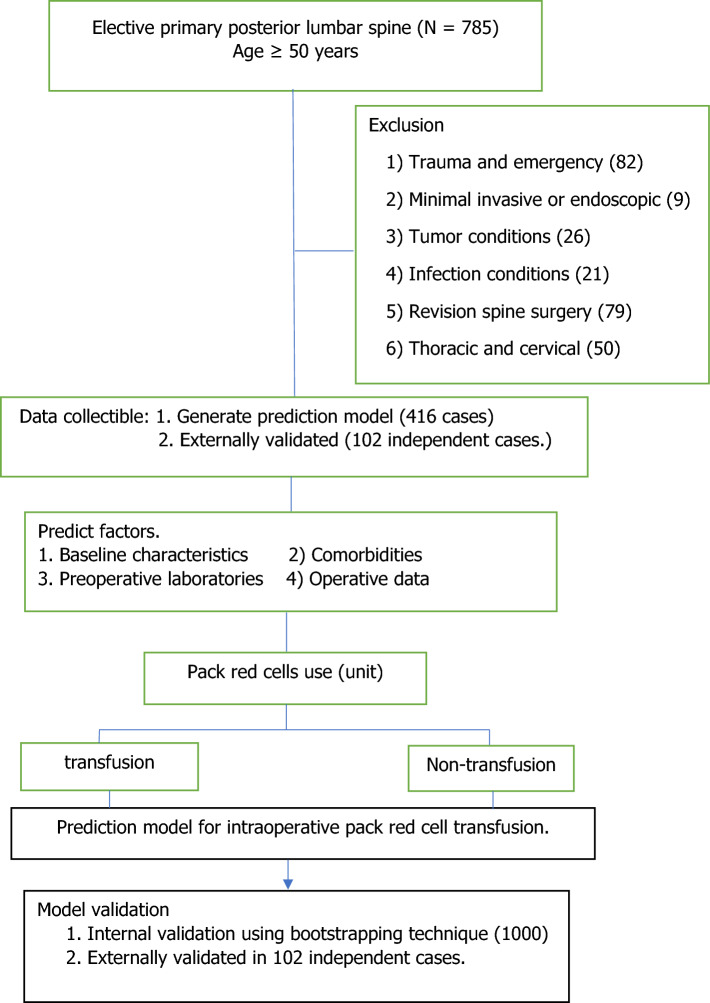
Flow diagram of patient enrollment.

Among the 785 patients identified, a total of 518 patients met the criteria, including 416 patients (transfusion group, n = 178, 43% classified by 1–2 units was 34% and at least three units was 9%); nontransfusion group, n = 238, 57%) included in the analysis for developing the model, and the remaining 102 patients were included in the independent case in external validation. Of these, 267 patients were excluded because they underwent (1) trauma and emergencies (n = 82), (2) minimally invasive or endoscopic techniques(n = 9), (3) tumors (n = 26), (4) infection (n = 21), (5) revision spine surgery (n = 79), and (6) thoracic and cervical levels (thoracic and cervical spine fusion at the same time of lumbar spine fusion) (n = 50) (Fig. 1).

Baseline characteristics, preoperative laboratory results, and operative modality findings are shown in Table 1 (prediction model development cases) and Table 2 (externally validated in 102 independent cases). Prognostic factors with a high predictive performance showing a statistically significant *P*-value of < 0.05, AuROC of > 0.60 (select from Diagnostic Accuracy as the minor sufficient level)^23^, and clinically meaningful correlation were chosen. The univariable logistic regression analysis, which included the preoperative Hct cut-off of 38% (level suitable for blood donation)^24^, laminectomy, TLIF, and sacral fusion, were identified as critical clinical predictors.

**Table 1:** Comparison of the baseline characteristics of the PRC transfusion and non-transfusion groups (prediction model development cases).

PRC	Elective primary posterior lumbar spinal fusion				
	PRC transfusion (n = 178)	PRC non-transfusion (n = 238)	OR	*P-*value	AuROC (95% CI)
Female (%)	71.9	64.3	1.42	0.101	0.54 (0.49, 0.58)
Age (y) (mean ± SD)	62.4 ± 7.8	61.7 ± 7.8	1.01	0.378	0.53 (0.47, 0.58)
Body mass index (kg/m^2^) (mean ± SD)	26.0 ± 4.1	25.3 ± 3.8	1.05	0.058	0.55 (0.50, 0.61)
Diabetes mellitus type II (%)	20.8	14.7	1.52	0.106	0.53 (0.49, 0.57)
Anticoagulant/antiplatelet use (%)	9.0	5.0	1.86	0.117	0.52 (0.49, 0.54)
Hypertension (%)	53.4	51.3	1.09	0.670	0.51 (0.46, 0.56)
Pulmonary disease (%)	1.7	3.8	0.44	0.218	0.51 (0.47, 0.50)
ASA classification (%)	1.4 ± 0.5	1.2 ± 0.5	1.97	0.001	0.59 (0.54, 0.63)
Preoperative Hct level (mean ± SD)	37.3 ± 3.8	39.0 ± 3.4	0.87	< 0.001	0.64 (0.31, 0.42)
Preoperative platelet count (× 10^3^/mL) (mean ± SD)	273.9 ± 66.3	285.9 ± 76.8	1.00	0.097	0.54 (0.40, 0.52)
Operative time (min) (mean ± SD)	254.7 ± 60.8	183.6 ± 53.3	1.02	< 0.001	0.81 (0.77, 0.85)
Laminectomy (level) ± SD	3.2 ± 0.8	1.9 ± 0.8	5.87	< 0.001	0.85 (0.82, 0.89)
Fusion method (level) (mean ± SD)					
PL fusion	3.5 ± 1.0	2.2 ± 1.0	3.51	< 0.001	0.81 (0.77, 0.85)
TLIF	0.7 ± 0.8	0.3 ± 0.5	2.60	< 0.001	0.64 (0.59, 0.69)
PLIF	0.0 ± 0.2	0.1 ± 0.3	0.44	0.153	0.52 (0.47, 0.49)
Sacrum fusion (%)	81.5	26.5	12.20	< 0.001	0.78 (0.73, 0.82)
Pedicular screw (level) (mean ± SD)	3.6 ± 0.9	2.2 ± 1.1	3.51	< 0.001	0.81 (0.78, 0.85)
Tranexamic acid (%)	75.3	57.6	2.25	< 0.001	0.59 (0.54, 0.63)
Estimate blood loss (mL) (EBL mean ± SD)	1281.8 ± 996.5	476.7 ± 254.9	1.00	< 0.001	0.87 (0.83, 0.90)

ASA, American Society of Anesthesiologists; AuROC, area under the receiver operating characteristic; EBL, estimate blood loss; Hct, hematocrit; PL fusion, posterolateral fusion; TLIF, transforaminal lumbar interbody fusion; OR, odds ratio; PLIF, posterior lumbar interbody fusion; PRC, packed red cells; *P* < 0.05, significant difference; 95% CI, 95% confidence interval.

**Table 2:** Comparison of the baseline characteristics of the PRC transfusion and non-transfusion groups (externally validated in 102 independent cases).

PRC	Elective primary posterior lumbar spinal fusion				
	PRC transfusion (n = 36)	PRC non-transfusion (n = 66)	OR	*P-*value	AuROC (95% CI)
Female (%)	72.2	59.1	1.80	0.190	0.57 (0.47, 0.66)
Age (y) (mean ± SD)	63.9 ± 8.2	64.3 ± 9.4	0.99	0.809	0.50 (0.38, 0.62)
Body mass index (kg/m^2^) (mean ± SD)	25.6 ± 4.3	24.5 ± 3.3	1.09	0.132	0.58(0.45, 0.70)
Diabetes mellitus type II (%)	13.9	12.1	1.17	0.798	0.51 (0.43, 0.58)
Anticoagulant/antiplatelet use (%)	8.3	9.1	0.91	0.897	0.50 (0.43, 0.55)
Hypertension (%)	55.6	62.1	0.76	0.519	0.53 (0.36, 0.57)
Pulmonary disease (%)	5.6	3.0	1.88	0.536	0.51 (0.47, 0.56)
ASA classification (%)	1.6 ± 0.5	1.3 ± 0.6	2.28	0.039	0.60 (0.51, 0.71)
Preoperative Hct level (mean ± SD)	36.4 ± 4.5	38.8 ± 3.3	0.83	0.004	0.70 (0.18, 0.42)
Preoperative platelet count (× 10^3^/mL) (mean ± SD)	271.8 ± 82.1	294.5 ± 61.3	0.99	0.120	0.61 (0.27, 0.51)
Operative time (min) (mean ± SD)	250.1 ± 55.4	191.0 ± 56.7	1.02	< 0.001	0.79 (0.70, 0.88)
Laminectomy (level) ± SD	3.0 ± 0.8	1.8 ± 0.7	9.63	< 0.001	0.87 (0.79, 0.94)
Fusion method (level) (mean ± SD)					
PL fusion	3.7 ± 1.0	2.0 ± 0.9	5.74	< 0.001	0.86 (0.78, 0.94)
TLIF	0.8 ± 0.9	0.3 ± 0.6	2.19	0.007	0.63 (0.53, 0.73)
Sacrum fusion (%)	72.2	19.7	10.60	< 0.001	0.76 (0.67, 0.85)
Pedicular screw (level) (mean ± SD)	3.4 ± 0.9	1.9 ± 1.0	6.02	< 0.001	0.86 (0.79, 0.93)
Tranexamic acid (%)	66.7	62.1	1.22	0.648	0.52 (0.42, 0.62)
Estimate blood loss (mL) (EBL mean ± SD)	1211.9 ± 668.7	373.3 ± 176.7	1.01	< 0.001	0.93 (0.90, 0.98)

ASA, American Society of Anesthesiologists; AuROC, area under the receiver operating characteristic; EBL, estimate blood loss; Hct, hematocrit; PL fusion, posterolateral fusion; TLIF, transforaminal lumbar interbody fusion; OR, odds ratio; PLIF, posterior lumbar interbody fusion; PRC, packed red cells; *P* < 0.05, significant difference; 95% CI, 95% confidence interval.

**Figure 2:**
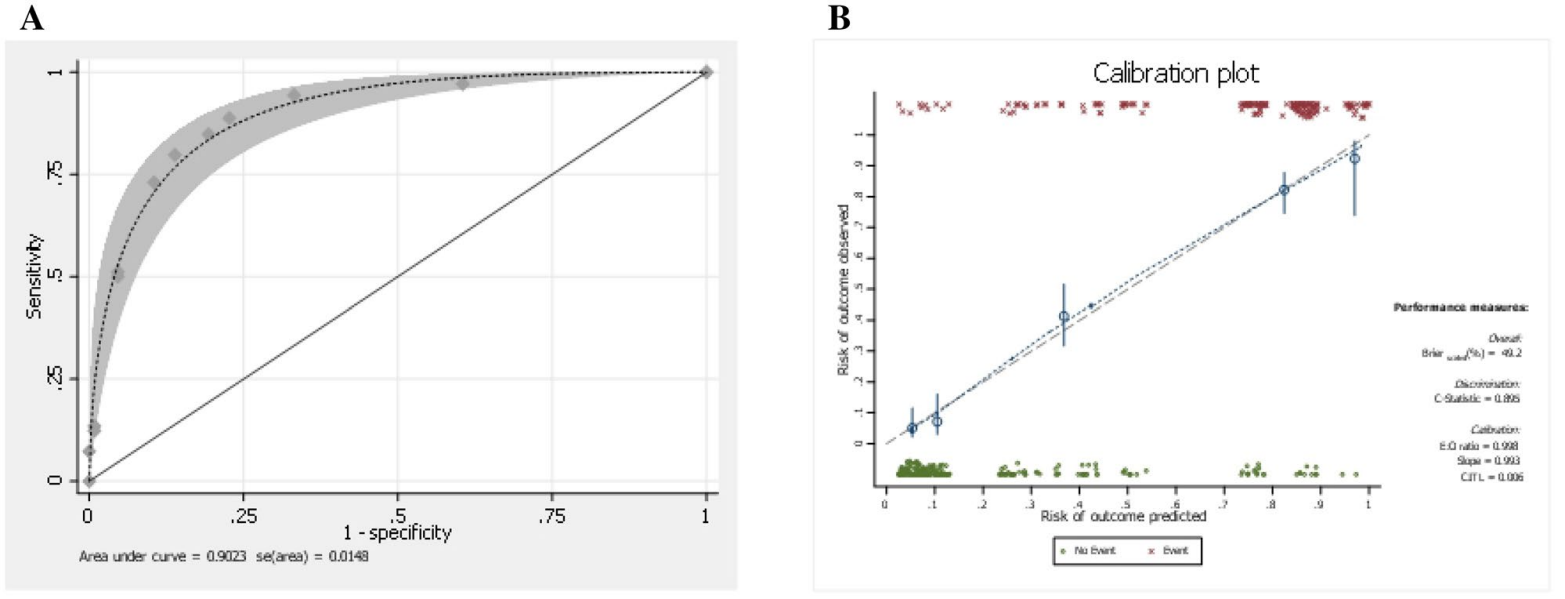
(**A**) Receiver operating characteristics (ROC) curves of the clinical prediction model for PRC transfusion. PRC, packed red cells. (**B**) Calibration plot of the model-predicted risk vs. the observed risk of using a PRC transfusion in primary elective lumbar spinal fusion. PRC, packed red cells.

The authors analyzed four potential clinical predictors using multivariable logistic regression (Table 3). The PRC transfusion sum score was calculated by adding the scores of each variable (sum score = preoperative Hct [score] + laminectomy [level] [score] + TLIF [level] [score] + sacral fusion [score]). This study transformed the model predictor (β) regression coefficients into simple scores. Subsequently, the authors developed a simplified model that incorporated clinically relevant factors that can be easily used in clinical practice. The model could predict the use of PRC transfusion with good discriminative ability (AuROC: 0.90 (95%CI 0.87, 0.93)) (Fig. 2A). The model correctly classified with sensitivity, specificity, PPV, and negative predictive values of 79.78%,86.13%, 81.14%, and 85.06%, respectively.

**Table 3:** Best multivariable clinical predictors.

Predictors	OR	95% CI	*P*-value	Beta coefficient	Adjusted βeta coefficient	Score
Preoperative hematocrit level						
≥ 38	1.00	reference	–	–	–	0
< 38	2.06	1.19, 3.59	0.010	0.73	1.00	1
Laminectomy (level)						
≤ 2	1.00	reference	–	–	–	0
> 2	10.41	5.91, 18.34	< 0.001	2.34	3.23	3
TLIF (level)						
≤ 1	1.00	reference	–		–	0
> 1	8.41	2.53, 27.92	0.001	2.13	2.93	3
Sacral fusion						
no	1.00	reference	–		–	0
yes	5.31	2.96, 9.51	< 0.001	1.67	2.30	2.5

OR, odds ratio; 95% CI, 95% confidence interval; β, logistic regression beta coefficient.

Adjusted βeta coefficient = βeta coefficient in that Raw/lowest βeta coefficient (*).

Preoperative planning procedure: laminectomy (level), TLIF (level), Sacral fusion.

sum score = preoperative hematocrit (score) + laminectomy (level) (score) + TLIF (level) (score) + sacral fusion (score).

Measures of calibration: The calibration plot showed that the model-predicted risk and observed risk of PRC transfusion concomitantly increased (C-statistic = 0.895, slope = 0.993) (Fig. 2B). Internal validation performance of the model via nonparametric receiver operating characteristics (ROC) with 1,000 bootstrap sampling techniques (bootstrap shrinkage = 0.993) and external validation in 102 independent cases (AuROC: 0.91, 95% CI 0.86, 0.97).

**Figure 3:**
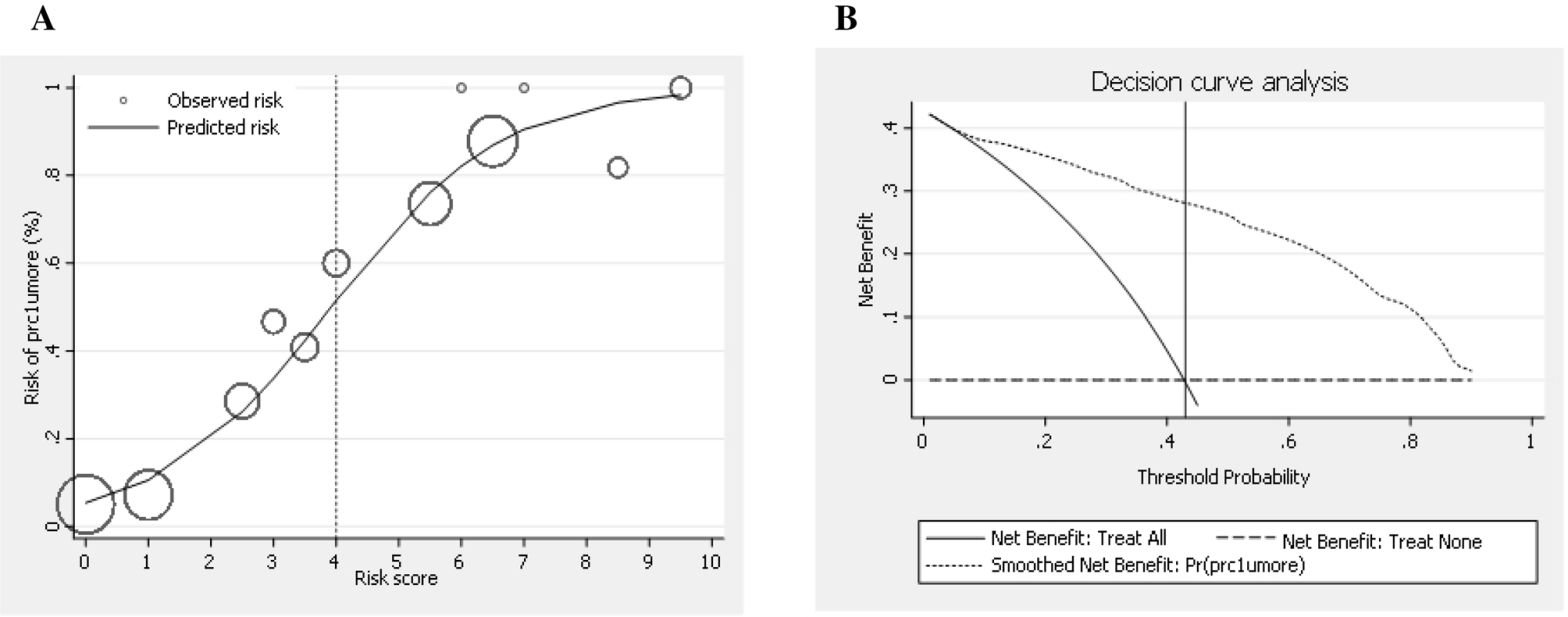
(**A**) Evaluation of the model performance in terms of clinical predictive ability. Observed risk (circle) vs. model-predicted risk (solid line) of PRC transfusion. The circle size represents the frequency of PRC transfusions in each score. PRC, packed red cells; prc1umore: use of PRC transfusion ≥ 1 unit. (**B**) Evaluation of the model performance in terms of clinical usefulness based on the score calibration curve and decision curve analysis. PRC, packed red cells; prc1umore, use of PRC transfusion ≥ 1 unit.

A model performance with a high-risk score (> 4) predicted PRC transfusion (Fig. 3A). The clinical predictions were categorized into two risk groups. The PPVs in the low-risk (≤ 4) and high-risk (> 4) groups were 18.4 (95% CI 13.9, 23.6) and 83.9 (95% CI 77.1, 89.3) respectively (Table 4).

**Table 4:** Distribution of prediction scores into low- and high-probability categories.

Score categories	Score	PRC transfusion (n = 178)		PRC nontransfusion (n = 238)		PPV (%)	95% CI	*P*-value
		n	(%)	n	(%)			
Low	≤ 4	48	(18)	213	(82)	18.4	(13.9, 23.6)	< 0.001
High	> 4	130	(84)	25	(16)	83.9	(77.1, 89.3)	< 0.001

PPV, positive predictive value; PRC, packed red cells; 95% CI, 95% confidence interval.

Model performance regarding clinical usefulness and curve analysis can explain the prediction model’s net benefit (NB) (PRC transfusion). A cut-off probability threshold of 0.43 (the prevalence point) indicated that our predicted model showed an NB of 2.8 times compared with that without the predictive model (Fig. 3B).

## Discussion

Spinal fusion is a commonly performed surgery for degenerative lumbar spine disease, but it carries a risk of significant blood loss (0.5–2 L) and often requires blood transfusions^25^. Blood transfusion is the primary treatment for blood loss and anemia during surgery. Studies have found that PRC transfusions are frequently needed in adult spine fusion surgery^2,6^. The 43% transfusion rate found in this study was consistent with some previous research (40–81%)^2,13,15^, but considerably higher than others (5–32%)^9,12,14^. The spine referral center hospital faces challenges in managing PRC transfusions due to cost constraints and blood shortages. Accurate prediction of PRC transfusion risk is crucial to optimizing blood resource utilization and improving preoperative preparation^12^.

This study has four strengths in preoperative PRC transfusion prediction in elective lumbar spine fusion: (1) High accuracy: The model shows good discrimination ability and external validation (AuROC: 0.90 and 0.91, respectively). (2) Good calibration: The calibration slope is close to one, showing an exact prediction of the probability of PRC transfusion. (3) More accurate than previous models (Recent limited studies: Wang et al.^14^ proposed a prediction nanogram. The AuROC of this study was 0.898, which is the use of learning efforts for daily clinical practice. This study requires using parameters intraoperatively, making it impossible to predict preoperative PRC preparation. Nie et al.^15^, It was discriminatory (AuROC = 0.73), with a smaller sample size and only preoperative predictors. Previous studies indicated the substantial effect of intraoperative predictors^1,3,8,9,11–14,16^. Another previous study^3^ did not define the type of lumbar spine fusion is associated with variations in intraoperative blood loss. This model outperforms previous ones in predicting PRC transfusion because it uses preoperative procedural planning, a defined type of lumbar fusion, and is easy to apply. (4) Clinical application: Safely discussed to patients that preoperative blood preparation by a low score category is not an essential cross-match of PRC, and cross-match should be performed in 1–2 units of PRC following the suggestion from the MSBOS for a high-risk category^19^ (transfusion group, use 1–2 units was 79%). In cases where a physician decides to request more PRC units than the model recommends, it is essential to consider less expensive preparation types and screening methods to minimize costs.

The model uses four predictors to assess the risk of PRC transfusion: (1) A high preoperative Hct level indicates lower transfusion risk^11,13,26^. (2) Laminectomy at multiple levels: Increases bleeding due to more tissue detachment^8,9,11–13^. (3) TLIF at more than one level: Leads to increased bleeding in the intervertebral endplate^1,12^ and (4) Sacral fusion: More complex procedure with increased bleeding from muscle and soft tissue detachment^12^. This study has several limitations: (1) Needs further validation: The model needs to undergo a more comprehensive, prospective study to confirm its accuracy and reliability. (2) Retrospective design and missing data: The retrospective nature of the study and the lack of some data (small number of PLIF cases (more risk of nerve root injury than TLIF), bleeding disorders, surgeon experience, preoperative blood predictors (active partial thromboplastin time (APTT), prothrombin time (PT), and other clotting factors were not routine preoperative laboratory) limit the model's comprehensiveness. (3) Single-center study: The model needs external validation in other hospitals to ensure its generalizability. (4) Safety protocol: To prioritize patient safety, it is essential to develop a collaborative safety protocol with the blood bank to address potential errors in transfusion predictions and ensure prompt access to blood products if needed.

In conclusion, the present study developed a model for predicting the preoperative preparation for PRC. This model has high discriminative ability, simplicity, and cost-effectiveness. Further research is necessary for external validation in other spine referral center hospitals and a prospective study cohort with a large sample size before application.

## Data Availability

The datasets generated during and/or analyzed during the current study are available from the corresponding author on reasonable request.

## References

[1] Huang YH, Ou CY (2015). Significant blood loss in lumbar fusion surgery for degenerative spine. World Neurosurg..

[2] Blackburn CW (2019). Clinical outcomes associated with allogeneic red blood cell transfusions in spinal surgery: A systematic review. Glob. Spine J..

[3] Xu N (2022). Prospective study of preoperative autologous blood donation for patients with high risk of allogeneic blood transfusion in lumbar fusion surgery: A study protocol of a randomised controlled trial. BMJ Open.

[4] Hasan O (2018). "It’s a precious gift, not to waste”: is routine cross matching necessary in orthopedics surgery? Retrospective study of 699 patients in 9 different procedures. BMC Health Serv. Res..

[5] Narissirikul S, Thanapipatsiri S, Vanadurongwan B (2016). Evaluation of the effectiveness of preoperative blood ordering guideline in elective spine, knee replacement, and hip replacement surgery. J. Med. Assoc. Thai..

[6] Ristagno G (2018). Incidence and cost of perioperative red blood cell transfusion for elective spine fusion in a high-volume center for spine surgery. BMC Anesthesiol..

[7] Saringcarinkul A, Chuasuwan S (2018). Maximum surgical blood order schedule for elective neurosurgery in a University Teaching Hospital in Northern Thailand. Asian J. Neurosurg..

[8] Aoude A (2016). Incidence, predictors, and postoperative complications of blood transfusion in thoracic and lumbar fusion surgery: an analysis of 13,695 patients from the American College of Surgeons National Surgical Quality Improvement Program database. Glob. Spine J..

[9] Basques BA (2015). Risk factors for blood transfusion with primary posterior lumbar fusion. Spine.

[10] Stammers AH, Tesdahl EA, Mongero LB, Stasko A (2019). Gender and intraoperative blood transfusion: analysis of 54,122 non-reoperative coronary revascularization procedures. Perfusion.

[11] Choovongkomol C, Ariyanuchitkul T, Choovongkomol K (2021). Prevalence and associated factors of blood transfusion in spinal surgery at Maharat Nakhonratchasima hospital: A retrospective study. Thai J. Anesthesiol..

[12] Morcos MW (2018). Predictors of blood transfusion in posterior lumbar spinal fusion: A Canadian spine outcome and research network study. Spine.

[13] Miri M (2015). Predictive factors of blood loss and hospital stay in patients with major lumbosacral surgeries: A multi-center, prospective, cross-sectional survey. Arch. Neurosci..

[14] Wang H (2021). Establishment and assessment of a nomogram for predicting blood transfusion risk in posterior lumbar spinal fusion. J. Orthop. Surg. Res..

[15] Nie Z, Ma W, Hu J (2021). Models to predict the probability for intraoperative RBC transfusion during lumbar spinal stenosis and femoral fracture surgeries in aged patients. Transfus. Apher. Sci..

[16] Heard JC (2023). Predictors of blood transfusion in patients undergoing lumbar spinal fusion. World Neurosurg..

[17] Morbée L, Chen M, Herregods N, Pullens P, Jans LBO (2021). MRI-based synthetic CT of the lumbar spine: Geometric measurements for surgery planning in comparison with CT. Eur. J. Radiol..

[18] Raturi M, Kusum A (2020). The blood supply management amid the COVID-19 outbreak. Transfus. Clin. Biol..

[19] Newfoundland Labrador. Maximum surgical blood ordering schedule. *Department of Health and Community Services*. https://www.gov.nl.ca/hcs/files/bloodservices-pdf-max-surgical-blood-order.pdf (2021).

[20] Ehresman J (2020). Cost-benefit analysis of tranexamic acid and blood transfusion in elective lumbar spine surgery for degenerative pathologies. J. Neurosurg. Spine.

[21] Baeza-Delgado C (2022). A practical solution to estimate the sample size required for clinical prediction models generated from observational research on data. Eur. Radiol. Exp..

[22] Maiti T, Pradhan V (2009). Bias reduction and a solution for separation of logistic regression with missing covariates. Biometrics.

[23] Simundic AM (2012). Diagnostic accuracy, Part 1: Basic concepts. Point Care J. Patient Test Technol..

[24] American Red Cross. What does hematocrit mean?. *Hematocrit*. https://www.redcrossblood.org/donate-blood/dlp/hematocrit.html.

[25] Kang T (2019). Patient blood management during lumbar spinal fusion surgery. World Neurosug..

[26] Barrie U (2022). Transfusion guidelines in adult spine surgery: a systematic review and critical summary of currently available evidence. Spine J..

